# Human Cytomegalovirus Nuclear Capsids Associate with the Core Nuclear Egress Complex and the Viral Protein Kinase pUL97

**DOI:** 10.3390/v10010035

**Published:** 2018-01-13

**Authors:** Jens Milbradt, Eric Sonntag, Sabrina Wagner, Hanife Strojan, Christina Wangen, Tihana Lenac Rovis, Berislav Lisnic, Stipan Jonjic, Heinrich Sticht, William J. Britt, Ursula Schlötzer-Schrehardt, Manfred Marschall

**Affiliations:** 1 Institute for Clinical and Molecular Virology, Friedrich-Alexander University of Erlangen-Nürnberg, Erlangen 91054, Germany; eric.sonntag@uk-erlangen.de (E.S.); sabrina.wagner@uk-erlangen.de (S.W.); hanife.strojan@web.de (H.S.); christina.wangen@uk-erlangen.de (C.W.); 2 Department of Histology and Embryology, Faculty of Medicine, University of Rijeka, Rijeka 51000, Croatia; tihana.lenac@medri.uniri.hr (T.L.R.); berislav.lisnic@medri.uniri.hr (B.L.); stipan.jonjic@medri.uniri.hr (S.J.); 3 Division of Bioinformatics, Institute of Biochemistry, Friedrich-Alexander University of Erlangen-Nürnberg, Erlangen 91054, Germany; heinrich.sticht@fau.de; 4 Departments of Pediatrics and Microbiology, School of Medicine, University of Alabama at Birmingham, Birmingham, AL 35294, USA; wbritt@uab.edu; 5 Department of Ophthalmology, University Medical Center Erlangen, Erlangen 91054, Germany; ursula.schloetzer-schrehardt@uk-erlangen.de

**Keywords:** herpesviral nuclear egress, nuclear egress complex (NEC), viral protein kinase pUL97, immunogold-electron microscopy, NEC-capsid interaction, human cytomegalovirus

## Abstract

The nuclear phase of herpesvirus replication is regulated through the formation of regulatory multi-component protein complexes. Viral genomic replication is followed by nuclear capsid assembly, DNA encapsidation and nuclear egress. The latter has been studied intensely pointing to the formation of a viral core nuclear egress complex (NEC) that recruits a multimeric assembly of viral and cellular factors for the reorganization of the nuclear envelope. To date, the mechanism of the association of human cytomegalovirus (HCMV) capsids with the NEC, which in turn initiates the specific steps of nuclear capsid budding, remains undefined. Here, we provide electron microscopy-based data demonstrating the association of both nuclear capsids and NEC proteins at nuclear lamina budding sites. Specifically, immunogold labelling of the core NEC constituent pUL53 and NEC-associated viral kinase pUL97 suggested an intranuclear NEC-capsid interaction. Staining patterns with phospho-specific lamin A/C antibodies are compatible with earlier postulates of targeted capsid egress at lamina-depleted areas. Important data were provided by co-immunoprecipitation and in vitro kinase analyses using lysates from HCMV-infected cells, nuclear fractions, or infectious virions. Data strongly suggest that nuclear capsids interact with pUL53 and pUL97. Combined, the findings support a refined concept of HCMV nuclear trafficking and NEC-capsid interaction.

## 1. Introduction

Human cytomegalovirus (HCMV, family *Herpesviridae*) represents a major, worldwide-distributed human pathogen. Primary HCMV infection of the immunocompetent host frequently remains asymptomatic but severe disease can occur upon infection of immunonaïve and immunocompromised individuals, such as neonates, transplant recipients and cancer or AIDS patients [1]. HCMV pathogenicity is often directly linked with the efficiency of viral replication and both events are substantially determined by the balance of virus-host interaction [2]. In this regard, prominent examples of protein-protein interactions have been described including the formation of viral-cellular multiprotein complexes [3,4,5,6,7].

The fine-regulated process of herpesviral nuclear egress is a primary obstacle for the transition of nucleocapsids through the nuclear envelope and represents a rate-limiting step of viral replication efficiency. Nuclear egress is not mediated by passage through the nuclear pore complex (NPC) due to limitations in size (reviewed by [8,9,10,11,12,13,14,15]). Instead, a non-canonical pathway has been described on the basis of nuclear lamina distortion, primary envelopment at the inner nuclear membrane and a subsequent sequence of cytoplasmic steps including de-envelopment/re-envelopment and virion maturation. Important molecular details of how nuclear capsid egress through the nuclear envelope is achieved have been mechanistically elucidated recently for HCMV and other herpesviruses (reviewed by [11,12,15,16,17]).

As an initial step, HCMV pUL50 and pUL53 form a core nuclear egress complex (NEC) by heterodimerization at the nuclear rim, before they recruit further viral and cellular proteins. The main constituents of the HCMV-specific multi-component NEC, as determined by protein interaction studies particularly including proteomic analyses ([3] and references therein), are pUL97, p32/gC1qR, emerin, protein kinase C (PKC) and additional proteins. Among these, pUL97 is of particular importance, since its kinase activity is primarily responsible for nuclear lamina disassembly during the late phase of HCMV replication. Earlier studies showed the NEC association of pUL97 is mostly mediated in an indirect way through p32/gC1qR binding, thus bridging pUL97 to the pUL50-pUL53 core NEC [18]. In addition, cellular protein kinases were found NEC-associated, in particular, PKC and cyclin-dependent kinase 1 (CDK1), as identified by pUL50-specific co-immunoprecipitation [19,20]. Concerning the association of nuclear HCMV capsids with the NEC, however, the mechanism remained widely undefined to date. A number of capsid proteins have been discussed as possible candidates for mediating the contact with the NEC including major capsid protein (MCP) and smallest capsid protein (SCP) [21,22,23,24]. In addition, a number of further candidates are conceivable, such as the capsid portal protein, the capsid vertex-specific complex (CVSC), or other known capsid components (Table 1; references a: [25,26,27,28,29,30,31,32,33,34,35,36,37]; b: [15,38,39,40,41,42,43,44,45,46,47]; c: [48,49,50,51]). So far, it remains unclear whether the core NEC proteins themselves mediate capsid contacts or whether further proteins of the multi-component NEC may be involved. In any case, the current state of the art strongly suggests a docking of HCMV capsids to distinct sites of the nuclear envelope, termed as lamina-depleted areas (LDAs) [52,53,54]. The transition through these sites is characterized by the capsid budding within large inner nuclear membrane (INM) invaginations resulting in primary envelopment into the perinuclear space.

As LDAs appear to represent the preferred sites of HCMV nuclear capsid egress, the mode of specific rearrangement of the nuclear lamina in a locally restricted manner has recently attracted major interest of researchers and particularly lamina phosphorylation could be defined as an initial molecular trigger (reviewed by [55]). The site-specific phosphorylation of lamins, primarily effected by HCMV protein kinase pUL97, leads to a destabilization of the nuclear lamina. According to the current view, this process is not only induced by lamin phosphorylation but also subsequent phosphorylation-dependent regulatory events, such as proline cis/trans isomerization [56]. Very recently, we and others described the functional and structural properties of herpesviral egress proteins, i.e., the essential and conserved core NEC formed by pUL50 and pUL53 in the case of HCMV ([15] and references therein). A general hallmark of herpesviral core NECs is their specific recruitment of further NEC-associated proteins, which in part are identical, in other parts, however, differ between individual herpesviruses [3,5,15]. Importantly, the herpesviral multi-component NEC has at least three different functional properties, i.e., (i) the reorganization of the nuclear lamina through the activity of recruited effector proteins; (ii) the inner nuclear membrane interaction to initiate viral budding processes; and (iii) the postulated docking of NEC proteins to viral nuclear capsids (reviewed by [11,15,17]). In this report, we investigated the association of nuclear HCMV capsids with NEC proteins and budding sites at the nuclear lamina. Specifically, we provide evidence that the HCMV core NEC, especially its constituent pUL53, may directly associate with virions and, moreover, that the viral nuclear protein kinase pUL97 is found associated with the core NEC. The findings support a refined concept of the nuclear role of the HCMV-produced NEC, in particular by facilitating the targeting of capsids to nuclear envelope budding sites.

## 2. Materials and Methods

### 2.1. Cell Culture and HCMV Infection

Primary human foreskin fibroblasts (HFFs) were cultivated in minimal essential medium (Thermo Fisher Scientific, Waltham, MA, USA) containing 7.5% foetal calf serum, 350 μg glutamine per mL and 100 μg gentamicin per mL. For infection experiments, HFFs (passage no. 10–15) were plated in 6-well plates or 175 cm^2^ cell culture flasks at a density of 3.2 × 10^5^ cells per well or 2–3 × 10^6^ cells per flask, respectively. One day later, HFFs were infected with either HCMV strain AD169 or recombinant HCMV strain AD169-GFP at multiplicities of infection (MOI) between 1.0 and 2.0 as previously described [57].

### 2.2. Antibodies

A description of the antibodies used in this study, i.e., for the various techniques of Western blot (WB), immunoprecipitation (IP), in vitro kinase assay (IVKA), immunogold labelling in electron microscopy (IEM), or further applications, is provided by Table 2 [3,6,19,20,22,24,56,58,59,60,61,62,63,64,65,66,67,68,69,70,71,72,73,74,75,76].

### 2.3. Immunogold Labelling and Transmission Electron Microscopy (TEM)

HFFs, plated on permanox coverslips (Thermo Fisher Scientific), were infected with HCMV strain AD169 at a MOI of 2.0. At 72 h post-infection (hpi), cells were fixed with 4% paraformaldehyde and 0.1% glutaraldehyde in 0.1 M cacodylate buffer (pH 7.4) for 10 min at room temperature. Cells were sectioned by using an ultramicrotome (Ultracut E; Leica Microsystems, Wetzlar, Germany). The thickness of sections to be further analysed was 30 to 40 nm. For post-embedding immunogold labelling, fixed cells were rinsed in buffer, dehydrated serially to 70% ethanol at −20 °C and embedded in LRWhite resin (Electron Microscopy Sciences, Hatfield, PA, USA). Ultrathin sections were successively incubated in Tris-buffered saline (TBS), 0.05 M glycine in TBS, 0.5% ovalbumin and 0.5% fish gelatine in TBS, primary antibodies diluted in TBS-ovalbumin overnight at 4 °C and finally in 10 nm gold-conjugated secondary antibodies (Biocell, Cardiff, UK) diluted 1:30 in TBS-ovalbumin for 1 h. Primary antibodies included: rabbit monoclonal antibody (mAb)-Lamin A/C (EPR4100; Abcam plc, Cambridge, UK; 1:30 dilution in PBS), rabbit polyclonal antibody (pAb)-pS22-lamin A/C (Cell Signaling Technology, Danvers, MA, USA; 1:5 dilution), rabbit pAb-UL97 (Boston, kindly provided by Dr. D. Coen, Boston, MA, USA; 1:1000) and mouse pAb-UL53 (Bologna, kindly provided by Dr. P. Dal Monte, Bologna, Italy; 1:200). After rinsing, sections were stained with uranyl acetate and examined with a transmission electron microscope (906E, Zeiss Microscopy, Oberkochen, Germany). For each antibody staining, the numbers of analysed sections were five to ten (each carrying approx. 50 cells) and numbers of capsid clusters were between one to five per infected cell nucleus. In negative control experiments, the primary antibody was replaced by PBS or equimolar concentrations of nonimmune rabbit IgG or an irrelevant primary antibody.

### 2.4. Co-immunoprecipitation (CoIP) Assay

Two 175 cm^2^ cell culture flasks of HCMV-infected HFFs per sample were used for protein-protein interaction experiments utilizing CoIP. Immunoprecipitation from whole cell lysates (WCL) was performed three days post-infection (dpi) under previously described conditions using 2 µL of mouse mAb-UL50 (50.01) or mAb-SCP (11-2-23). In addition, CoIP experiments were performed using cell-free virions (VIR) as input. To this end, viral particles from virus-containing supernatants of HFFs infected with HCMV strains AD169, TB40, Merlin, or Epstein-Barr virus (EBV) strain B95-8 were pelletized by a 3 h centrifugation step at 4 °C and 20,800 rcf. Subsequently, virus pellets were lysed in 500 µL CoIP buffer (50 mM Tris/HCl (pH 8.0), 150–300 mM NaCl, 5 mM EDTA, 0.5% NP-40, 1 mM PMSF, 2 mg aprotinin mL^−1^, 2 mg leupeptin mL^−1^ and 2 mg pepstatin mL^−1^) and lysates were used for CoIP as described previously [77]. In every case, CoIP samples and expression controls taken prior to the addition of CoIP antibody (i.e. input) were subjected to standard Western blot analysis using mouse mAbs as follows: mAb-MCP (28-4), mAb-SCP (11-2-23), mAb-UL97 (97.01), mAb-UL50 (UL50.01) and mAb-UL53 (53.01) (produced by the authors of this study; see Table 2).

### 2.5. In Vitro Kinase Assay (IVKA)

Association of viral pUL97 kinase activity with nuclear HCMV capsids was determined in vitro after cell fractionation and immunoprecipitation of HCMV capsids. Therefore, the nuclear fraction of two 175 cm^2^ cell culture flasks of HCMV-infected HFFs per sample was isolated at 4 dpi using CST Cell Fractionation Kit #9038 (Cell Signaling Technology) according to the manufacturer’s protocol. HCMV capsids were immunoprecipitated from the isolated nuclear fraction with 2 µL of mAb-SCP and subjected to IVKA reaction using 5 µCi of [γ-^33^P] adenosine triphosphate (ATP) as described previously [77,78]. Finally, samples were prepared for separation by 12.5% SDS-PAGE, followed by transfer to nitrocellulose membrane (A. Hartenstein, Würzburg, Germany) for Western blotting. Autoradiographic membranes were exposed to a phosphorimager plate and measured using a CR 35 Bio phosphorimager (raytest Isotopenmessgeräte GmbH, Straubenhardt, Germany).

## 3. Results

### 3.1. Association of HCMV Capsids with the Core NEC Constituent pUL53 Visualized by Immuno-Gold Electron Microscopy

**Figure 1:**
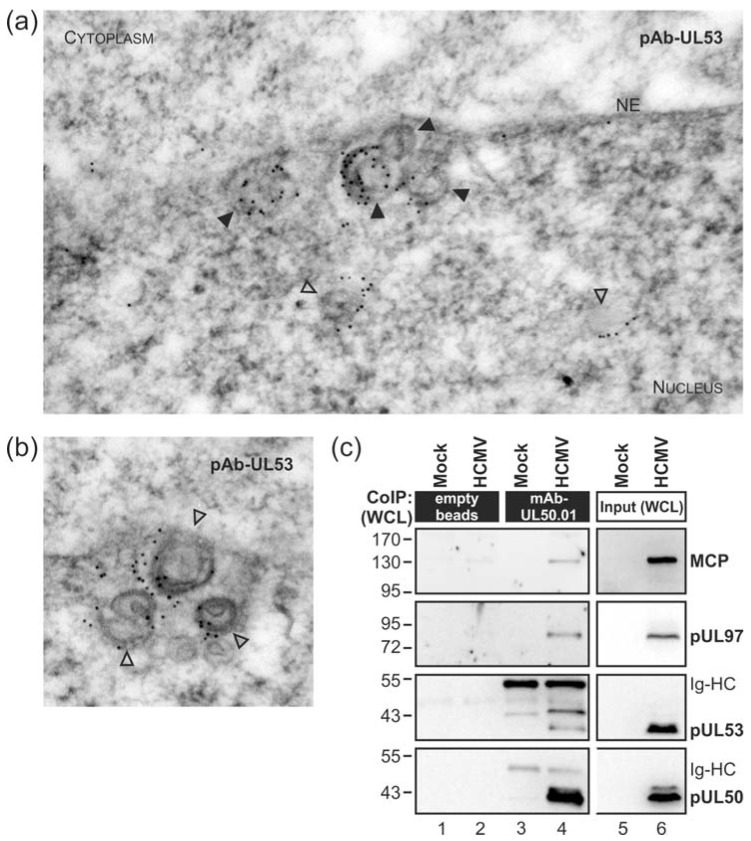
Association of the viral core nuclear egress complex (NEC) constituent pUL53 with intranuclear human cytomegalovirus (HCMV) capsids and capsids budding at the inner nuclear membrane (INM). (**a**,**b**) HCMV-infected primary human foreskin fibroblasts (HFFs) were harvested at 3 dpi and subjected to immunogold staining of viral pUL53. Samples were analysed by transmission electron microscopy (TEM), 35,970-fold magnification. NE, nuclear envelope; open arrowheads, intranuclear HCMV capsids; filled arrowheads, HCMV capsids budding at nuclear membranes; (**c**) Co-immunoprecipitation (CoIP) analysis with HCMV-infected whole cell lysates (WCL). HCMV- or uninfected (mock)-HFFs were lysed at 4 dpi followed by immunoprecipitation with monoclonal antibody (mAb)-UL50.01 coupled to protein A sepharose (lanes 3–4) or empty beads as a CoIP negative control (lanes 1–2). CoIP samples (lanes 1–4) and expression control samples (input, representing one-twentieth of CoIP samples; lanes 5–6) were subjected to Western blot analysis using protein-specific antibodies. Ig-HC, cross-reactive band for immunoglobulin heavy chain; MCP, major capsid protein.

The nuclear egress of HCMV capsids is regulated by the multifunctional NEC. However, the question as to how nuclear capsids are recruited to the NEC and to specific nuclear budding sites has not been answered yet. In order to address the intranuclear fine-localization of HCMV egress proteins, we performed immunostaining of viral proteins in ultrathin sections of infected-cell material evaluated by electron microscopy (immuno-EM). Primary human foreskin fibroblasts (HFFs) were infected with HCMV strain AD169 at a multiplicity of infection (MOI) of 2 and viral capsids were visualized at budding sites of the nuclear envelope at 3 days post-infection (dpi) (Figure 1a,b). Immuno-EM staining pointed to the association of viral pUL53, as a core NEC component, with nuclear capsids. The signal patterns strongly suggested a tight association of pUL53 with capsids currently budding at nuclear membranes (Figure 1a, filled arrowheads; 91% of analysed membrane-associated capsids were coated with gold particles) and even at times prior to reaching the nuclear envelope (Figure 1a,b, open arrowheads; 17% of intranuclear capsids were coated with gold particles). The specificity of the used primary and secondary antibodies has been proven in control stainings of mock-infected and HCMV-infected cells, respectively (Figure S1). This finding of pUL53-capsid interaction was reminiscent of a recent report on herpes simplex virus type 1 (HSV-1) capsid interaction with the pUL53 homolog pUL31, mainly based on fluorescence microscopic analyses [79].

**Figure 2:**
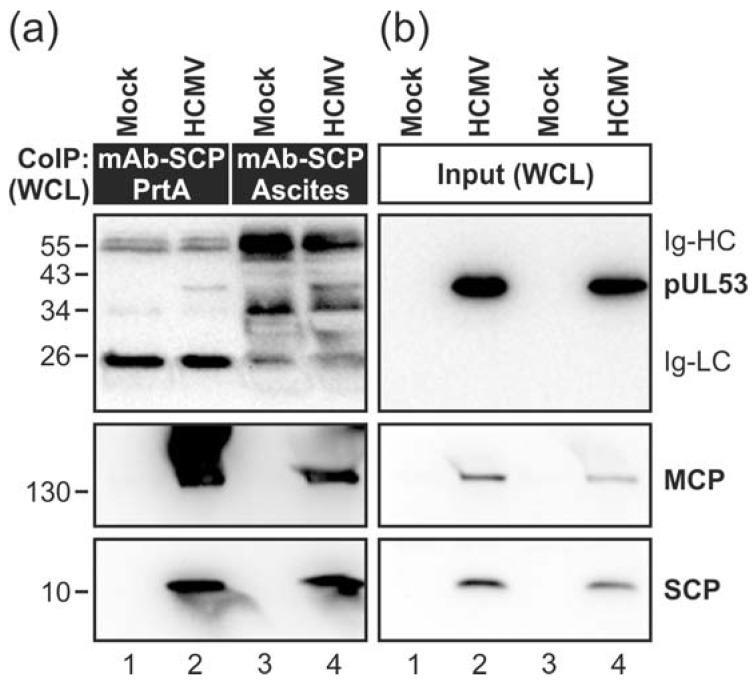
Interaction of HCMV capsid proteins SCP and MCP with the core NEC constituent pUL53. HFFs were infected with recombinant HCMV strain AD169 expressing the green fluorescent protein (GFP) or remained uninfected (mock) as indicated. Cells were lysed at 4 dpi followed by immunoprecipitation with a monoclonal antibody (mAb) against SCP produced as a protein A purified tissue culture supernatant (mAb-SCP PrtA; lanes 1–2) or ascites fluid (mAb-SCP Ascites; lanes 3–4). Co-immunoprecipitates (**a**) and expression control samples (input; (**b**)) were subjected to Western blot analysis using protein-specific antibodies. SCP, smallest capsid protein; MCP, major capsid protein; Ig-HC and Ig-LC, cross-reactive band for immunoglobulin heavy and light chains.

In accordance with the EM data, the major capsid protein (MCP) was co-immunoprecipitated with the core NEC and the NEC-associated protein kinase pUL97 from HCMV-infected whole cell lysates (WCL) using a pUL50-specific antibody (i.e. mAb-UL50.01; Figure 1c). These findings were further supported by performing additional co-immunoprecipitation (CoIP) experiments. Using two different preparations of mAb-SCP, MCP was massively detected in the co-precipitate (Figure 2, middle panels). In addition, a minor quantity of pUL53 could also be detected (Figure 2a, upper panel). The low level of pUL53 found in the CoIP fraction with SCP might reflect the fact that only viral capsids budding at nuclear membranes showed a constant pUL53 association in the immunogold labelling electron microscopy (EM) analysis. It should, however, be noted as an important indication that pUL53 might be the first contact point for nuclear capsids when they approach the viral NEC.

### 3.2. HCMV Kinase pUL97 Associates with Viral Capsids Already in the nucleus of Infected Cells

**Figure 3:**
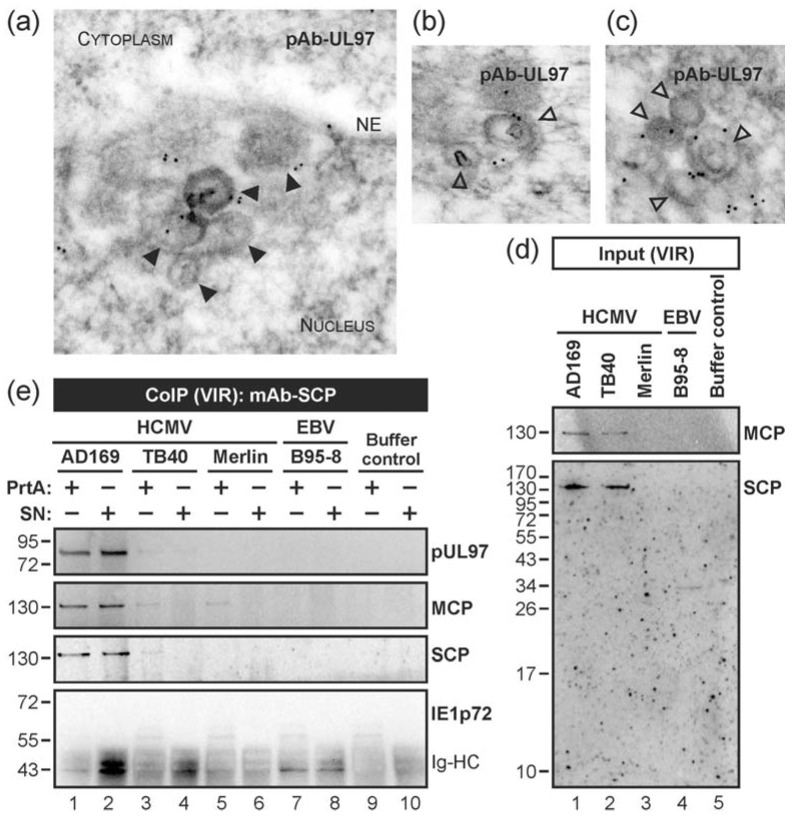
Association of the HCMV-encoded protein kinase pUL97 with viral capsids in the nucleus. (**a**–**c**) HCMV-infected HFFs were harvested at 3 dpi and subjected to immunogold staining of viral pUL97. Samples were analysed by TEM, 35,970-fold magnification. NE, nuclear envelope; open arrowheads, intranuclear HCMV capsids; filled arrowheads, HCMV capsids budding at nuclear membranes; (**d,e**) CoIP analysis with cell-free virions (VIR) as input. Virions from HCMV strains AD169, TB40 or Merlin (harvested from identical quantities of HFF producer cell layers, i.e., one T175 flask each) were lysed followed by CoIP using protein A-purified mAb-SCP (PrtA) or unpurified supernatant of mAb-SCP hybridoma cultures (SN). EBV strain B95-8 or buffer served as CoIP negative controls ((**d**) lanes 4–5; (**e**) lanes 7–10). CoIP samples (**e**) and expression control samples (input; (**d**)) were both subjected to Western blot analysis using antibodies against the indicated proteins. Note, no band was detected at the molecular weight of approximately 72 kDa using an antibody directed against immediate early protein 1 (IE1p72; (**e**), lower panel) pointing to the specificity of the CoIP. Ig-HC, cross-reactive band for immunoglobulin heavy chain.

The possibility that capsid association of pUL53 may also include pUL97 (Figure 1c), which is a multifunctional regulator that phosphorylates a number of substrates including pUL50 and pUL53 [20], prompted us to address further questions about the functional consequences of these protein interactions. To this end, we asked whether the protein kinase pUL97, particularly responsible for the major regulatory events in nuclear lamina rearrangement during viral egress, is likewise detectable by EM-based localization studies as a protein associated with nuclear capsids. In fact, a staining pattern of nuclear capsid co-localization was observed for pUL97 similar to pUL53 (although at some lower detectable quantity than pUL53, i.e., approx. 33% of evaluated capsids were found pUL97-associated; Figure 3a). The signal distribution suggests an interaction of pUL97 with the surface interface of variable stages of nuclear particles (Figure 3b,c). The specificity of the antibody staining was again analysed using mock-infected and HCMV-infected cells (Figure S2). Notably, the control images point to a specific detection of pUL97 in this EM analysis.

Next, we confirmed the EM-based evidence of pUL97-capsid interaction by performing CoIP using protein lysates from virion stocks of the HCMV strains AD169, TB40 and Merlin. In this setting, mAb-SCP was able to co-immunoprecipitate both MCP and pUL97 (Figure 3d,e). For pUL97, the most prominent signals of interaction were obtained for strain AD169 (Figure 3e, lanes 1–2), which may relate to the high titre of this viral stock. It should be mentioned that SCP is highly conserved among the three strains, i.e., >95% identity on amino acid level (with a single amino acid exchange of SCP Merlin at position V68I). Interestingly, SCP—detectable with a molecular weight of approx. 12 kDa in control lysates (see Figure 2, lanes 2 and 4)—migrated at a high molecular size of ≥130 kDa, even larger than MCP, in the input and immunoprecipitates from virion lysates (Figure 3d, lanes 1–2 and Figure 3e, lanes 1–3). One possible explanation would be a tightly linked packaging of SCP together with MCP in virions, which is not separable by standard denaturating SDS-PAGE. We confirmed the detectability of this high molecular form of SCP when we used whole cell lysates of HCMV-infected cells for CoIP with monoclonal antibodies against three different structural proteins, i.e., mAb-MCP, mAb-pp150 and mAb-gB. Note that recent structural investigations proved the tight association of pp150 with capsids, particularly a pp150 cysteine tetrad–to-SCP interaction [23] (which might possibly not be completely resolved by SDS-PAGE). In our supplemental experiment, all three mAbs proved to be active in CoIP under these conditions and produced co-immunoprecipitates between MCP, pp150 and SCP (while gB appeared to be mostly separated by the use of detergents in the CoIP lysis buffer). Specifically, for SCP two forms were detected, namely the small 12-kDa SCP and an inseparable high molecular weight form ≥130 kDa, which might represent a covalent conjugate either with pp150 or a so far undefined other protein (Figure S3).

**Figure 4:**
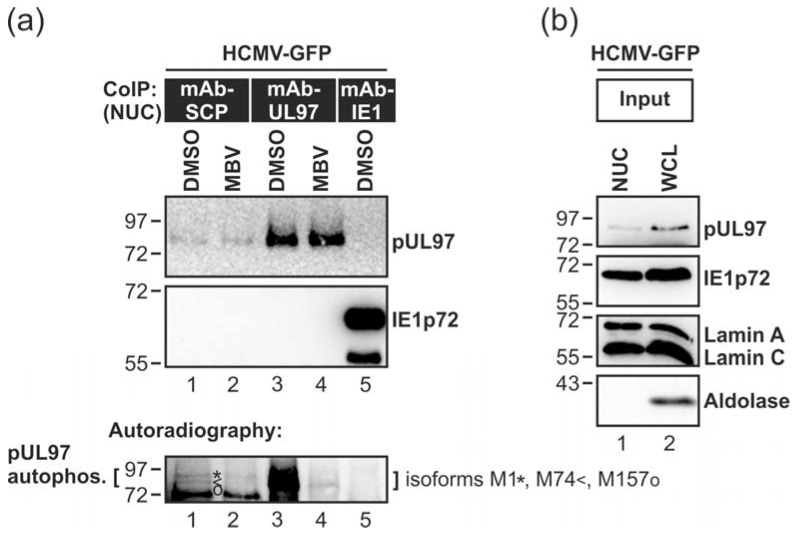
The kinase activity of capsid-associated pUL97 purified from the nucleus of HCMV-infected cells. HFFs were infected with recombinant HCMV AD169-GFP and treated with the pUL97 kinase inhibitor maribavir (MBV) or the solvent dimethylsulfoxide (DMSO). At 4 dpi, cells were harvested and the nuclear fraction (NUC) was isolated from whole cell lysates (WCL). The nuclear fraction was used for immunoprecipitation of HCMV-encoded proteins SCP, pUL97, or IE1p72 as a control. Precipitates were subjected subsequently to a pUL97-specific in vitro kinase assay (IVKA). ((**a**), upper panels CoIP) Prior to IVKA reactions, the quality of CoIP samples was verified by Western blot analysis. ((**a**), lower panel Autoradiography) IVKA reactions were performed under conditions optimized for pUL97 activity and signals of autophosphorylation are depicted. Note the labelling of the three known isoforms of pUL97, M1, M74 and M157, which all possess autophosphorylation activity; (**b**) The input levels of all relevant proteins were stained on parallel Western blot panels.

In order to substantiate the potential interaction between pUL97 and HCMV capsids, we investigated if nucleocapsid-associated pUL97 exerts kinase activity. For this purpose, nuclear extracts were prepared from HCMV-infected fibroblasts and pUL97 was co-immunoprecipitated with nuclear HCMV capsids by the use of mAb-SCP. An assessment of pUL97 autophosphorylation activity was performed by in vitro kinase assays (IVKA). The results indicated capsid-associated activity of pUL97 (Figure 4a, lane 1; see pUL97 autophosphorylation, lower panel). The three previously characterized isoforms of pUL97, all known to possess autophosphorylation activity [67], were labelled to illustrate the specificity of the bands. The autoradiography shows a reduction of pUL97 autophosphorylation activity (Figure 4a, lane 1, lower panel) by pUL97 inhibitor MBV (Figure 4a, lane 2, lower panel).

Maximal activity of pUL97 was monitored by the use of mAb-UL97 (Figure 4a, lanes 3–4) and the specificity of the reaction was controlled by mAb-IE1, not showing any background signal of pUL97 activity (Figure 4a, lane 5; for expression levels of viral proteins and the success of nuclear extraction, i.e., separation of nuclear lamins A/C from cytoplasmic aldolase, are shown in Figure 4b). This result argues for very early binding of catalytically active pUL97 to nuclear stages of viral capsids that may be required for subsequent steps of nuclear egress regulation. Note that this IVKA result may provide a first argument for pUL97-capsid interaction, either through direct or indirect contact possibly involving further NEC proteins.

### 3.3. Targeted Mode of Lamina Disassembly by Capsid-Associated pUL97

**Figure 5:**
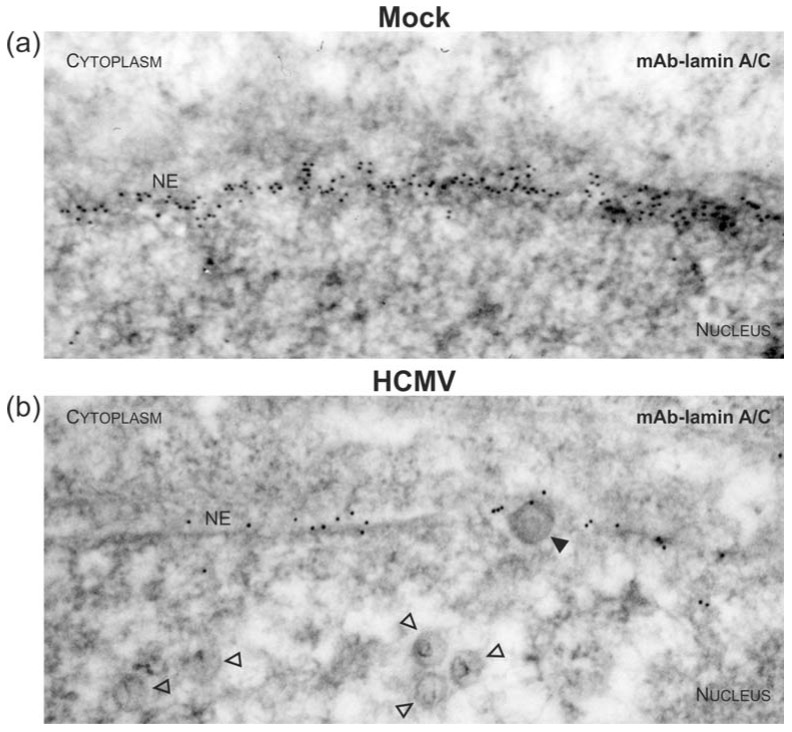
Disassembly of the nuclear lamina during nuclear egress of HCMV capsids visualized by electron microscopy. HFFs were infected with HCMV strain AD169 (**b**) or remained uninfected (mock; (**a**)) as indicated. Cells were harvested at 3 dpi and subjected to immunogold staining of A-type lamins (i.e. lamin A/C). Samples were analysed by TEM, 35,970-fold magnification. NE, nuclear envelope; open arrowheads, intranuclear HCMV capsids; filled arrowheads, HCMV capsids budding at nuclear membranes.

**Figure 6:**
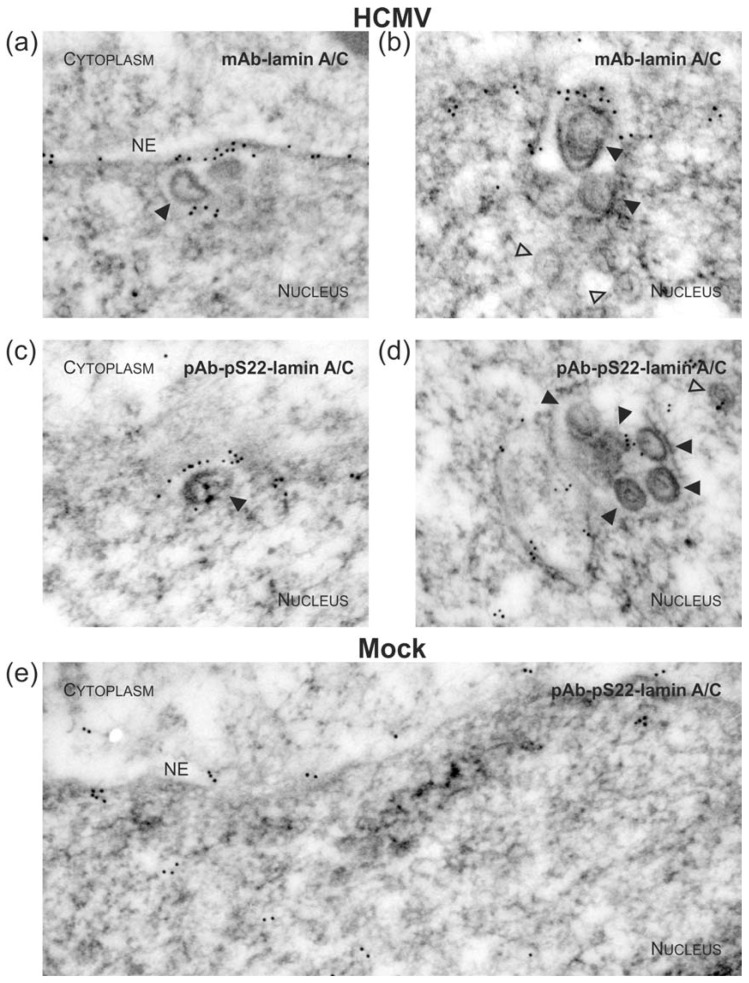
Accumulation of phosphorylated nuclear lamins at INM-budding sites of HCMV capsids during nuclear egress. HCMV-infected (**a**–**d**) or uninfected (mock; (**e**)) HFFs were stained with phosphorylation-independent (**a**,**b**) or phosphorylation-dependent (**c**–**e**) lamin A/C primary antibodies and gold-tagged secondary antibodies. Samples were analysed by TEM, 35,970-fold magnification. NE, nuclear envelope; open arrowheads, intranuclear HCMV capsids; filled arrowheads, HCMV capsids budding at nuclear membranes.

To visualize virus-induced phosphorylation of the nuclear lamina, which is mainly mediated by pUL97 kinase activity [4,18,52,56,80,81], monoclonal or polycolonal antibodies (mAb/pAb) against nuclear lamin A/C were used for immuno-EM, either recognizing lamins in a phosphorylation-independent or phosphorylation-dependent manner (Figure 5, Figure 6 and Figure S4). As previous reports demonstrated a major site of lamin phosphorylation at position serine 22, pAb-pS22-lamin A/C was applied in this analysis (Figure 6c–e) and was compared to signals obtained for overall lamin A/C detection with mAb-lamin A/C (Figure 5 and Figure 6a,b). The homogeneous rim-like distribution of lamin A/C signals observed in uninfected cells (Mock, Figure 5a and Figure S4b) was drastically altered in HCMV-infected cells; i.e., a thinning of lamin A/C-specific immuno-EM grains was detected (Figure 5b and Figure S4a), which corresponds to the earlier description of nuclear lamina thinning and the induction of LDAs [52,54]. The typical pattern of lamin redistribution in HCMV-infected cells includes lamina thinning and the formation of lamina-depleted areas. However, it was surprising to detect that despite of the low concentration of lamina-associated gold particles at the nuclear envelope of HCMV-infected cells, budding events were exclusively found at sites which were still decorated with lamin A/C , (Figure 5b and Figure 6a, filled arrowheads). In fact, staining with pAb-pS22-lamin A/C suggested that this portion of lamin A/C is still phosphorylated at serine 22 (Figure 6c and Figure S4e) which is a prerequisite for lamin dissociation from the nuclear envelope. Interestingly, a similar association of lamins, stained with either mAb-lamin A/C or the phospho-specific pAb-pS22-lamin A/C, was observed with capsids adjacent to intranuclear membranes (Figure 6b,d). These structures resemble previously described large tubular INM infoldings which were postulated to be utilized for nuclear egress of human and mouse cytomegalovirus (MCMV) capsids [53]. Taken together, it is intriguing to speculate that nuclear capsids not only interact with the viral core NEC and pUL97 but also with nuclear lamins, which undergo site-specific phosphorylation during the process of nuclear egress in a capsid/NEC-associated manner.

## 4. Discussion

**Figure 7:**
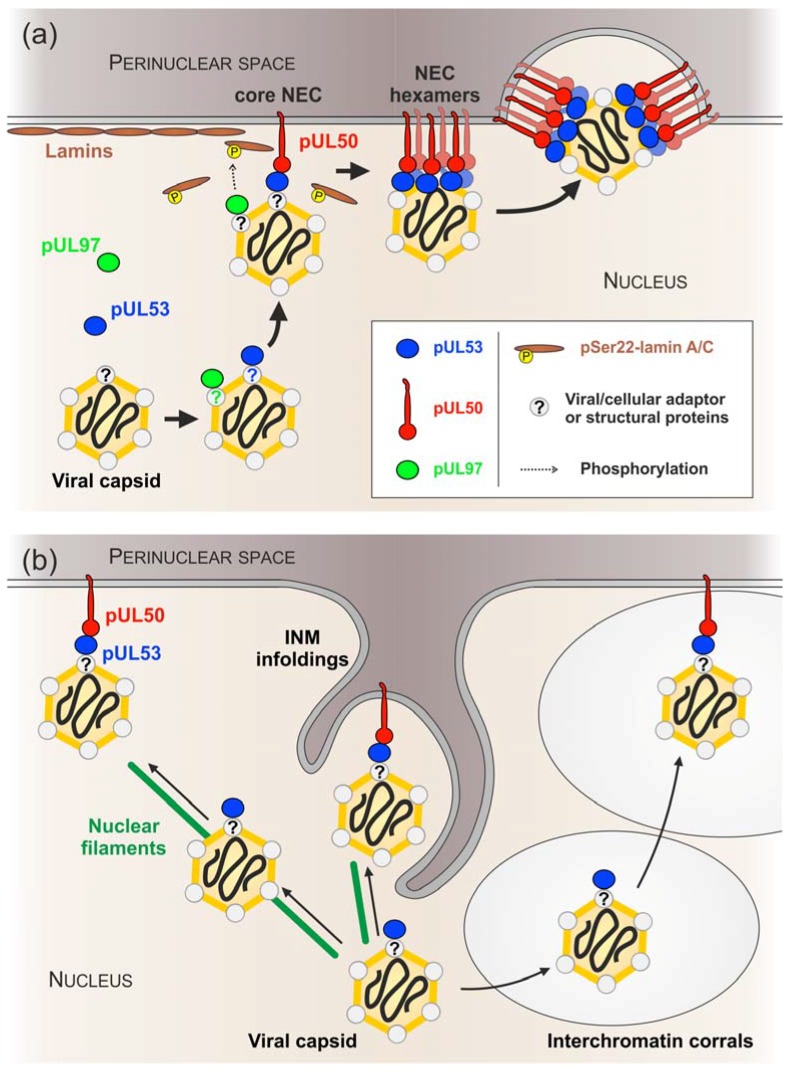
Schematic presentation of theoretical options that may lead to the recruitment of HCMV capsids to INM budding sites. (**a**) First, core NEC component pUL53 and NEC-associated protein kinase pUL97 bind to viral capsids in the nucleoplasm. Binding might be mediated directly via structural viral proteins (e.g., MCP, SCP, pUL85, or CVSC proteins) or indirectly via viral or cellular adaptor proteins. Second, pUL53-coated capsids dock to membrane-anchored pUL50 under formation of the core NEC. Capsid-associated pUL97 might be responsible for phosphorylation of remaining lamin A/C molecules providing access to the INM. Hexameric core NEC assemblies finally initiate budding into the perinuclear space; (**b**) Depiction of putative mechanisms of how HCMV capsids reach the INM are depicted on the basis of evidence mainly provided by this study and studies with alpha-herpesviruses. Black solid arrows define optional directions of capsid transport/trafficking. See description of individual possibilities in the main text. Diagrams not to scale.

A series of recent reports addressed questions about the regulation of HCMV nuclear egress orchestrated by the HCMV-specific core NEC forming a multi-component viral-cellular NEC extension reviewed by [15]. However, the association of the NEC with HCMV nuclear capsids remained enigmatic in several aspects. Our study focused on experimental approaches illustrating the association of viral capsids with the HCMV-specific core NEC and viral protein kinase pUL97 (Figure 7). The central findings were as follows: (i) nuclear HCMV capsids may directly associate with NEC proteins at viral budding sites of the nuclear lamina; (ii) the core NEC constituent pUL53 and viral protein kinase pUL97 showed, at least to some extent, intranuclear interaction with virions; (iii) in vitro kinase assays provided initial evidence for kinase activity of capsid-associated pUL97; and (iv) immune-EM staining patterns using phospho-specific and phospho-independent lamin A/C antibodies are compatible with our earlier postulate of a targeted mode of virion egress at lamina-depleted areas.

Data presented in this study on the association of pUL53 with intranuclear HCMV capsids appear to be congruent with previous reports using HSV-1 and pseudorabies virus (PRV), in which it was demonstrated that the core NEC of these two alpha-herpesviruses binds to viral capsids [82,83,84]. In particular, the PRV and HSV-1 pUL31 proteins, homologs of HCMV pUL53, were identified to be enriched on purified viral particles and pUL31-capsid binding was shown to be independent of its core NEC partner pUL34 [82,83]. Consistent with this, we observed HCMV pUL53 associated with viral capsids in the nucleus of infected cells by EM immunogold staining. Interestingly, this was not restricted to capsids located at the nuclear envelope, presumably budding into the perinuclear space but pUL53 coating was also observed on capsids which had not yet reached the INM. Co-immunoprecipitation analyses confirmed that pUL53, as well as its core NEC partner pUL50, is strongly suggested to be bound to the structural capsid proteins MCP and SCP. In a previous study, we identified MCP and the HCMV triplex protein pUL85 associated with pUL50-pUL53 when analysing complexes from HCMV-infected cells by mass spectrometry [3]. As all these proteins, MCP, SCP and pUL85, represent structural components, they may provide a direct link between nuclear viral particles and the core NEC. In previous studies, we and others showed that pUL50 is targeted to the INM, probably exclusively due to the function of its transmembrane domain [78,85,86,87]. The immunogold-EM data presented in this study suggests that capsid binding may be mediated by the pUL53 component of the core NEC. In line with this, based on the crystal structures of the core NECs of HCMV, HSV-1 and PrV [88,89,90,91], a conserved alpha-helix in pUL53 at the membrane-distal end of the NEC was proposed to mediate binding to the capsid [11,14].

To this end, it remains to be elucidated whether an additional viral or cellular capsid-associated protein may support this interaction. Notably, candidate proteins for the recognition of capsids by the core NEC were recently proposed for HSV-1 [83,84]. The HSV-1 homolog of pUL53 binds to a complex of the HSV-1-encoded proteins pUL17 and pUL25 [83]. These proteins, together with a third HSV-1 protein, i.e., pUL36, belong to a heterotrimeric complex located exclusively at the vertices of the icosahedral herpesviral capsid, which is therefore termed capsid vertex-specific complex (CVSC) [25]. Due to preferential loading of the CVSC on DNA-containing capsids (termed C capsids), binding of the core NEC to the CVSC might provide the mechanistic basis of a quality control to assure nuclear egress of only mature C capsids. The general importance of the CVSC for HSV-1 nuclear egress was at least shown for pUL25 [92]. In the absence of pUL25, neither primary enveloped particles in the perinuclear space nor capsids in the cytoplasm were observed by transmission electron microscopy (TEM), although all capsid types, including C capsids, were regularly formed and even were detected frequently adjacent to the INM [92]. In the case of HCMV, there is conflicting evidence as to whether the homologous CVSC proteins, pUL77 and pUL93, can interact with one of the core NEC proteins [93,94].

The presented data furthermore suggests that at least a portion of pUL53, which is not yet incorporated into the NEC located at the INM, associates with HCMV capsids somewhere in the nucleoplasm. It is tempting to speculate that pUL53-coated capsids might subsequently reach the INM to dock to membrane-bound pUL50. This is consistent with the recently revealed mechanism of capsid transport to the INM based on free diffusion through chromatin-devoid areas (i.e. interchromatin corrals) enlarged in herpesvirus-infected cells (Figure 7b) [95,96]. It is worthy of note that final capsid docking at the INM might require hexameric assemblies of the core NEC as postulated recently [15]. In particular, according to the recent crystal structures, the HCMV core NEC forms hexameric ring-like arrangements [88]. The stoichiometry and size of these rings matches that of the hexon-ordered arrangements of the major capsid protein. For this reason, it seems plausible that both hexameric structures, i.e., NEC and capsids, may directly interact with each other during nuclear egress [15]. However, such an interaction would also imply that the monomeric building blocks of these structures, e.g., pUL53 and an individual capsid protein, may additionally be capable to interact with each other, although with much lower affinity.

This point was strongly illustrated by our theoretical estimate, which generally suggests an increase of affinity resulting from multivalent interactions compared to monomeric interactions. We speculated whether monomeric pUL53 interaction with capsids might occur at a lower affinity than multivalent interaction of the hexameric pUL50-pUL53 NEC with capsids. For this purpose, an inspection of the literature was compiled in Table 3 references [97,98,99,100,101,102,103,104,105,106,107]. Most of the examples refer to protein-ligand interactions that gain affinity upon multimerization. For example, the Sos1 protein contains five proline-rich motifs that interact with the two SH3 domains of Grb2. The effective dissociation constant (K_d_) for the formation of a Sos1-Grb2 complex is 100 times smaller than the smallest K_d_ for the binding of a single Grb2 SH3 domain to a proline-rich motif on Sos1 [99]. In addition, there are also several examples demonstrating that this principle similarly applies to the interaction of proteins with lipids, sugars, or DNA (Table 3). For example, the multimeric protein S binds with at least 250-fold higher affinity to phospholipid bilayers compared to monomeric protein S [97]. For the methyl-CpG-binding domain (MBD) protein, which recognizes methylated CpG sites in double-stranded DNA, a covalently linked tetramer binds DNA more than 50 fold tighter than a monomeric MBD [98]. The gain in affinity for the multimeric interactions ranges from one to five orders of magnitude depending on the nature and stoichiometry of the interaction. The highest gain in affinity (>10^4^ fold) is observed for a pentameric compared to a monomeric ligand binding to the pentameric heat-labile enterotoxin (Table 3). From all systems referenced, the stoichiometry is closest to that of a putative hexamer-hexamer interaction between HCMV core NEC and capsid. Thus, we propose that a hexameric compared to a monomeric kind of NEC-capsid interaction would cause a rather large gain in affinity that might even exceed the values reported in Table 3.

Moreover, immunogold-EM confirmed previous studies based on confocal microscopy, which stated that local disassembly of the nuclear lamina was induced in HCMV-infected cells [4,18,52,80]. However, it was surprising to see that the remaining lamin A/C was enriched at capsid budding sites of the nuclear envelope. The use of a phospho-dependent antibody indicated that these lamin A/C molecules are already phosphorylated at serine 22 and, therefore, are likely to be dissociated from the residual nuclear lamina [56,108]. Consistent with this, we hypothesized that the HCMV protein kinase pUL97 may also bind to capsids in the nucleus (Figure 7a). It is known that pUL97, being a component of the herpesvirus-characteristic tegument layer, is packaged into HCMV particles, which are released from the host cell [109,110]. However, to date, it is not clear whether a substantial quantity of pUL97 already associates with HCMV capsids in the nucleus or in a later step during maturation in the cytoplasm. Thus, it remains to be elucidated whether pUL97 is permanently associated with capsids during nuclear egress and cytoplasmic maturation or whether nuclear capsid-binding of pUL97 is transient and final tegumentation of pUL97 takes place at a later stage in the cytoplasm.

## 5. Conclusions

In conclusion, we provide novel insights into the specific mechanisms and protein interactions relevant to HCMV nuclear egress. In particular, data are consistent with the idea that the core NEC constituent pUL53 may be involved in the transport of newly assembled HCMV capsids from the nucleoplasm to the nuclear envelope (Figure 7a). It remains to be determined whether this process really involves an active recruitment of capsids along nuclear filaments such as F-actin that, in addition, may essentially be dependent on pUL53. Alternatively, it seems suggestive that nuclear envelope targeting of capsids may be mediated by crossing interchromatin corrals by Brownian motion in the end connected with the capsids’ pUL53-pUL50 core NEC interaction. In both cases, current data suggests that budding into the perinuclear space can either occur at the nuclear envelope or even at large INM infoldings (Figure 7b). Future studies will have to provide more molecular data to confirm this concept and to clarify further details of the process.

## Figures and Tables

**Table 1:** Herpesviral nuclear capsid-associated proteins.

Type of Protein	Alpha-Herpesvirus ^a^ HSV-1	Beta-Herpesvirus ^b^ HCMV	Gamma-Herpesvirus ^c^ EBV
**Capsid portal**	pUL6	pUL104	BBRF1
**Capsid scaffolding**	pUL26.5	pUL80.5/pUL80a	BdRF1
**CVSC**	pUL17	pUL93	BGLF1
	pUL25	pUL77	BVRF1
	pUL36	nd	nd
**Terminase complex**	pUL15	pUL89	BGRF1/BDRF1
	pUL28	pUL56	BALF3
	pUL33	pUL51	BFRF1A
**Cleavage-packaging**	pUL32 ^d^	pUL52 ^d^	BFLF1 ^d^
**NEC core protein**	pUL31	pUL53 ^e^	nd
**Protein kinase**	nd	pUL97 ^e^	nd

^a^ References [25,26,27,28,29,30,31,32,33,34,35,36,37]; ^b^ references [38,39,40,41,42,43,44,45,46,47]; ^c^ references [48,49,50,51]; ^d^ capsid-association in question; ^e^ data of this study and references [3,15]; nd, not determined.

**Table 2:** Basic characteristics and specific reactivities of antibodies used in this study.

Designation	Type of Antibody *	Type of Antigen	Demonstrated Reactivity **	References
mAb-lamin A/C, (EPR4100)	mAb, rabbit	synthetic peptide, aa 500–600	WB, IF, IEM	[3,56,58]
pAb-pS22-lamin A/C (#2026)	pAb, rabbit	synthetic phosphopeptide, pS22	WB, IF, IEM	[3,59,60]
mAb-IE1p72 (P63-27)	mAb, mouse IgG	purified infected cell nuclei	WB, IF, IP	[61,62,63,64]
pAb-UL97 (Boston)	pAb, rabbit	baculovirus-expressed pUL97, full-length 1-707	WB, IF, IEM, IP, MS, IV	[6,65,66,67,68]
mAb-UL97 (97.01)	mAb, mouse kappa IgG1	baculovirus-expressed MBP-UL97, full-length 1-707	WB, IF, IP, MS, IV, EL	[6,63,69]
pAb-UL53 (Bologna)	pAb, mouse	bacterially expressed β-gal-UL53, full-length 1-292	WB, IF, IEM, IP, EL	[19,20,70]
mAb-UL53 (53.01)	mAb, mouse kappa IgG1	bacterially expressed pUL53, fragment 50-292	WB, IF, IP, EL	[3,19,20]
mAb-UL50 (50.01)	mAb, mouse kappa IgG1	bacterially expressed pUL50, fragment 1-181	WB, IF, IP, EL	[3,19,20]
mAb-MCP (28-4)	mAb, mouse	gradient-purified virions	WB, IF, IP, CA, EL	[22,24,71,72]
mAb-SCP (11-2-23)	mAb, mouse	gradient-purified virions	WB, IF, IP, IV, CA, EL	[22,73]
mAb-pp150 (36-14/XPA)	mAb, mouse	bacterially expressed pp150/pUL32	WB, IF, IP, CA, EL	[74,75]
mAb-gB (27-287)	mAb, mouse	bacterially expressed gp58/gB	WB, IF, IP, EL	[63,76]

* mAb, monoclonal antibody; pAb, polyclonal antibody; ** WB, Western blot; IF, immunofluorescence staining; IEM, immunogold labelling in electron microscopy; IP, immunoprecipitation; MS, mass spectrometry-based proteomics (IP with protein complexes); IV, IVKA (in vitro kinase assay/IP); CA, capsid-binding applications (IP with capsids); EL, ELISA (enzyme-linked immunosorbent assay).

**Table 3:** Published examples of increased affinities resulting from multivalent types of protein-ligand interaction.

First Binding Partner	Second Binding Partner	Type of Complex Formed	Gain in Affinity	References
S protein	Phospholipid bilayer	Multimeric protein S	>250 fold	[97]
MBD protein	DNA CpG sites	Tetrameric MBD	>50 fold	[98]
Grb SH3 domain	Proline-rich motif of SOS	Two SH3 domains and five proline-rich motifs	>100 fold	[99]
ZAP70 SH2 domain	ITAM motifs of Syk kinase	Two SH2 domains and two ITAM motifs	>100 fold	[100]
HPV E6 protein	Peptidic ligand	Bivalent peptidic ligand	>300 fold	[101]
Lectins	Sugar ligands	Multimeric lectins and ligands	1–3 orders of magnitude	[102]
Staphylococcal protein A	Affibody	Dimeric affibody	3 orders of magnitude	[103]
Monoclonal IgG I	Fcγ receptors RIIA and RIIIB	Dimeric IgG I	200–800 fold	[104]
Receptor tyrosine kinase VEGFR-2	Peptidic ligand	Dimeric peptidic ligand	6–500 fold	[105]
*E. coli* heat-labile enterotoxin (pentamer)	Galactose (monomer)	Five galactose moieties linked by a pentavalent scaffold	>10^4^ fold	[106,107]

## Supplementary Materials

Supplementary materials can be found at www.mdpi.com/1999-4915/10/1/35/s1.
