# Supplementary material for: Human Cytomegalovirus Nuclear Capsids Associate with the Core Nuclear Egress Complex and the Viral Protein Kinase pUL97
Source: Viruses. 2018 Jan 13;10(1):35. doi: 10.3390/v10010035 (PMC5795448; doi:10.3390/v10010035)
Supplement: Supplementary file 1 [file viruses-10-00035-s001.pdf]

Supplementary material

# Human Cytomegalovirus Nuclear Capsids Associate with the Core Nuclear Egress Complex and the Viral Protein Kinase pUL97

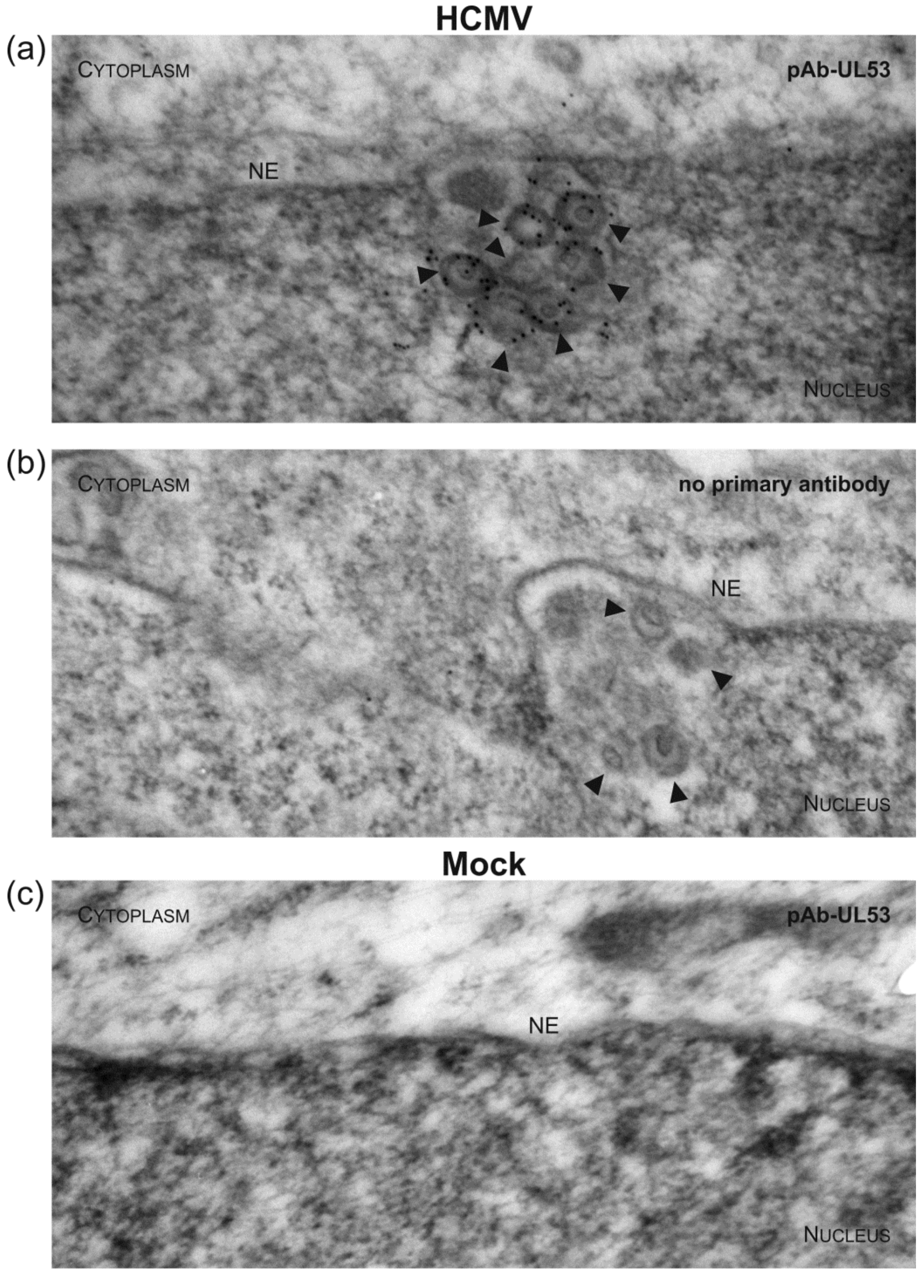


**Figure S1.** Specificity of the immunogold labelling of the HCMV-encoded nuclear egress protein pUL53. Primary human foreskin fibroblasts (HFFs) were infected with HCMV strain AD169 (**a**,**b**) or remained uninfected (mock; **c**). Cells were harvested at 3 dpi and subjected to immunogold staining of viral pUL53. Samples were analysed by transmission electron microscopy (TEM), 35,970-fold magnification. Note, pUL53 associates with capsids currently budding at nuclear membranes (**a**). The specificity of the immunogold staining was evaluated in HCMV-infected cells by using no primary antibody (**b**), and in uninfected control cells (**c**). In both cases, no background staining was detected. NE, nuclear envelope; filled arrowheads, HCMV capsids budding at nuclear membranes.

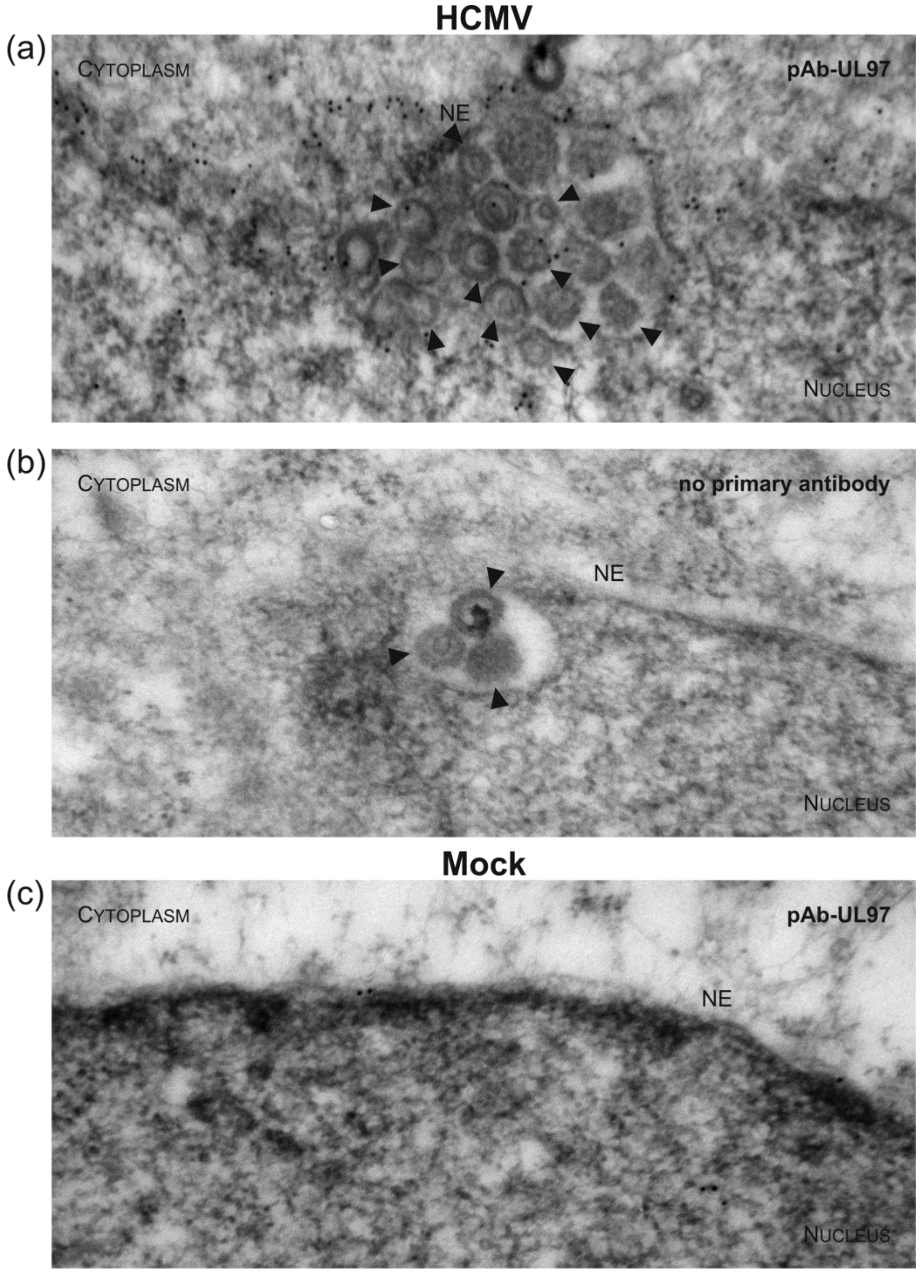


**Figure S2.** Specificity of the immunogold labelling of the HCMV-encoded protein kinase pUL97. Primary human foreskin fibroblasts (HFFs) were infected with HCMV strain AD169 (**a,b**) or remained uninfected (mock; **c**). Cells were harvested at 3 dpi and subjected to immunogold staining of viral pUL97. Samples were analysed by transmission electron microscopy (TEM), 35,970-fold magnification. Note, a subset of intranuclear capsids showed association with pUL97 (**a**). The specificity of the immunogold staining was evaluated in HCMV-infected cells by using no primary antibody, showing very low levels of background staining (**b**), and in uninfected control cells (**c**). NE, nuclear envelope; filled arrowheads, HCMV capsids budding at nuclear membranes.

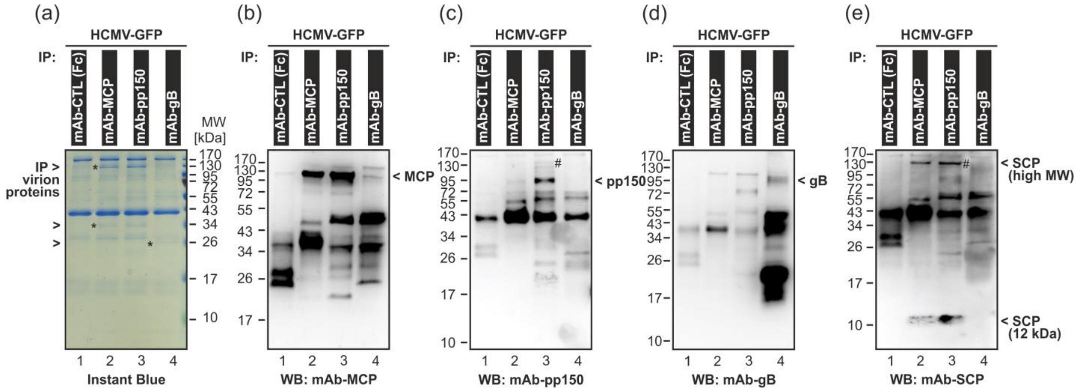


**Figure S3.** Co-immunoprecipitation (CoIP) demonstrating the protein characteristics and interaction properties of four different structural proteins of HCMV. HFFs were infected with HCMV AD169-GFP (MOI 0.5) for five days, harvested and lysed in CoIP buffer to be subjected to Dynabeads-based CoIP [Steingruber et al., 2016] using monoclonal antibodies against three different structural proteins, mAb-MCP, mAb-pp150 and mAb-gB. Immunoprecipitaes were separated on standard denaturating SDS-PAGE and processed for either direct total protein staining (a; Instant Blue; *, most abundant forms of immunoprecipitated virion proteins or fragments thereof) or for Western blot (WB) analysis. The WB detection antibodies were used as indicated in the bottom line (b-e), illustrating the successful immunoprecipitation of MCP, pp150, gB and SCP. Note, that CoIP signals were obtained for MCP, pp150 and SCP. SCP could be detected in two forms of low and high molecular weight (#).

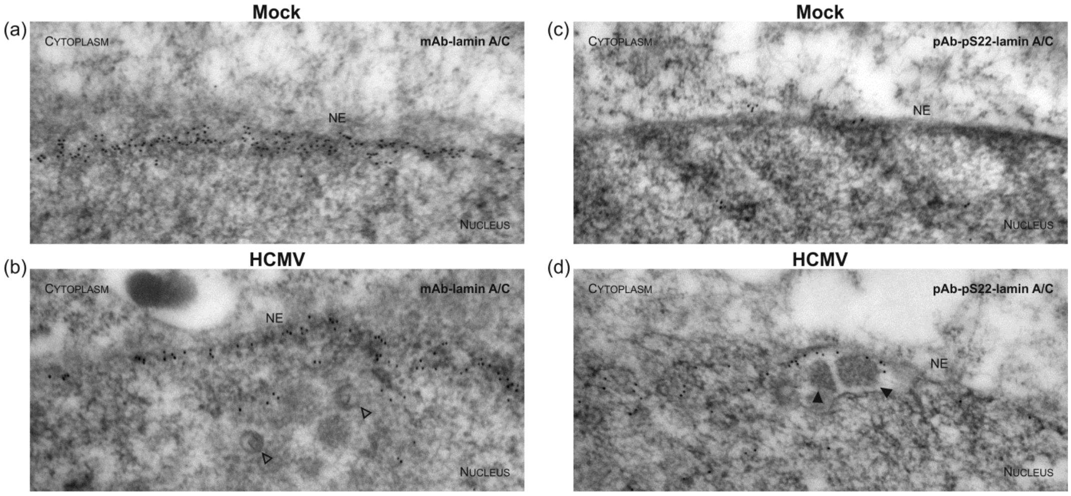


**Figure S4.** Altered distribution of nuclear lamin A/C at inner nuclear membrane-budding sites of HCMV capsids. Primary human foreskin fibroblasts (HFFs) were infected with HCMV strain AD169 (**b,d**) or remained uninfected (mock; **a,c**). Cells were harvested at 3 dpi and subjected to immunogold staining using phosphorylation-independent (**a,b**) or phosphorylation-dependent (**c,d**) lamin A/C primary antibodies and gold-tagged secondary antibodies. Samples were analysed by transmission electron microscopy (TEM), 35,970-fold magnification. NE, nuclear envelope; open arrowheads, intranuclear HCMV capsids; filled arrowheads, HCMV capsids budding at nuclear membranes.
